# Concurrent Statistical Learning of Ignored and Attended Sound Sequences: An MEG Study

**DOI:** 10.3389/fnhum.2019.00102

**Published:** 2019-04-17

**Authors:** Tatsuya Daikoku, Masato Yumoto

**Affiliations:** ^1^Department of Clinical Laboratory, Graduate School of Medicine, The University of Tokyo, Tokyo, Japan; ^2^Department of Neuropsychology, Max Planck Institute for Human Cognitive and Brain Sciences, Leipzig, Germany

**Keywords:** statistical learning, attention, auditory, Markov model, magnetoencephalography

## Abstract

In an auditory environment, humans are frequently exposed to overlapping sound sequences such as those made by human voices and musical instruments, and we can acquire information embedded in these sequences *via* attentional and nonattentional accesses. Whether the knowledge acquired by attentional accesses interacts with that acquired by nonattentional accesses is unknown, however. The present study examined how the statistical learning (SL) of two overlapping sound sequences is reflected in neurophysiological and behavioral responses, and how the learning effects are modulated by attention to each sequence. SL in this experimental paradigm was reflected in a neuromagnetic response predominantly in the right hemisphere, and the learning effects were not retained when attention to the tone streams was switched during the learning session. These results suggest that attentional and nonattentional learning scarcely interact with each other and that there may be a specific system for nonattentional learning, which is independent of attentional learning.

## Introduction

Statistical learning (SL) is a domain-general and automatic process that is innate to humans (Saffran et al., 1996; Perruchet and Pacton, 2006). By this process, the brain computes transitional probabilities (TPs) of sequential phenomena such as music and language without intention or awareness (Cleeremans et al., 1998), and incessantly updates acquired statistical knowledge to adapt to variable phenomena in environments (Daikoku et al., 2017c; Daikoku, 2018a,c,d).

Such SL effects have been observed in neurophysiological responses. For instance, the event-related potentials (ERPs) and magnetic fields (ERFs) represent a more sensitive method than behavioral responses (Schön and François, 2011; Paraskevopoulos et al., 2012; Koelsch et al., 2016). In a framework of predictive coding (Friston, 2005), when the brain codes TP distributions of a stimulus sequence, it expects a probable future stimulus with a high TP and inhibits the neural response to predictable external stimuli. Finally, the SL effects manifest as a difference in amplitudes between stimuli with lower and higher TPs. A body of studies detected SL effects on ERP/ERF such as P50 (Paraskevopoulos et al., 2012; Daikoku et al., 2016, 2017c; Daikoku and Yumoto, 2017), N100 (Sanders et al., 2002; Furl et al., 2011; Daikoku et al., 2014, 2015, 2017c), mismatch negativity (MMN; Koelsch et al., 2016; François et al., 2017; Moldwin et al., 2017), P200 (Cunillera et al., 2006; De Diego Balaguer et al., 2007; François and Schön, 2011; Furl et al., 2011), P300 (Batterink et al., 2015), and N400 components (Sanders et al., 2002; Cunillera et al., 2006, 2009; François and Schön, 2011; François et al., 2013, 2014). Compared with later auditory responses, the earlier auditory responses that peak at 20–80 ms (e.g., P50) have been attributed to parallel cortico-cortical or thalamo-cortical connections between the primary auditory cortex and the superior temporal gyrus (Adler et al., 1982). Thus, suppression of an early component of auditory responses to stimuli with a higher TP in lower cortical areas can be regarded as a transient expression of prediction error that is suppressed by predictions from higher cortical areas in a top-down connection (Skoe et al., 2015).

Most neurophysiological studies on SL have investigated the SL of single-tone sequences. In real-world auditory environments, however, humans are simultaneously exposed to overlapping sound sequences such as those made by musical instruments and human voices. Even when we selectively attend to the important information and ignore the unimportant information in overlapping sounds, humans generally acquire the information through both attentional and nonattentional processes (Jimenez and Castor, 1999; Aizenstein et al., 2004; Daikoku and Yumoto, 2017; Yumoto and Daikoku, 2018). However, few neurophysiological studies have examined attentional and nonattentional SL when learners are simultaneously exposed to multiple streams of sequences. To understand the mechanisms underlying SL, which is considered to occur automatically regardless of attention (Perruchet and Pacton, 2006), it is important to investigate how concurrent SL of attended and ignored sequences is reflected in neural responses.

In studies addressing consciousness during learning, the learning system has been divided into implicit learning, which may be accomplished through unconscious and nonattentional learning processes, and explicit learning, which may be accomplished through conscious and attentional learning processes (Reber, 1989; Ellis, 2005, 2009; Daikoku and Yumoto, 2017). The earlier studies suggested that explicit and implicit knowledge could be acquired by different learning processes and that explicit knowledge cannot be transformed into implicit knowledge through practice (Hulstijn, 2002). In contrast, other researchers have demonstrated that implicit and explicit knowledge can interact with each other (DeKeyser, 2003, 2007; De Jong, 2005; Ellis, 2005, 2009). Thus, interactive mechanisms between implicit and explicit learning remain a matter for debate (Krashen, 1982; Hulstijn, 2002; Daikoku et al., 2017b, 2018; Daikoku, 2018b).

To understand the neural mechanisms underlying concurrent attentional and nonattentional SL of auditory sequences, the present study used magnetoencephalography (MEG), a modality that can clearly resolve signals produced by the auditory cortices located bilaterally in the temporal lobes. We investigated how concurrent SL of simultaneous sequences of auditory stimuli is reflected in neuromagnetic responses and how the two forms of SL neurophysiologically interact with each other. MEG was recorded while participants listened to a dyad sequence (two-note chord). The dyad sequence can also be regarded as two types of auditory sequences consisting of low- and high-voice sequences in a distinct Markov-chain relationship. During the last third of each sequence, however, the Markov chains controlling the low and high voices were exchanged. The subjects were instructed to ignore one of the two types of sequences but to attend to the other. Given neural representations of SL effects, we hypothesized that if subjects could concurrently perform SL of the two sequences, a dyad that consisted of two frequent tones with higher TP should lead to the lowest response amplitudes, while a dyad that consisted of two rare tones with lower TP should lead to the highest response amplitudes. Furthermore, if the statistical knowledge of attended sequences and that of ignored sequences cannot be transformed from one type to the other, the SL effect should disappear when the sequential regulation of the high and low voices is exchanged in the final third of the sequence. In contrast, if statistical knowledge of the attended and ignored sequences can interact and be transformed, the SL effect should remain even when the sequential regulation of the low and high voices is exchanged.

## Materials and Methods

### Participants

Fifteen right-handed (57.9–100 in Edinburgh handedness, Oldfield, 1971) subjects without neurological and audiological disabilities participated (age range: 24–36 years, seven females, no absolute pitch). The present study was approved by the Ethics Committee of The University of Tokyo. All subjects were informed about this experiment including protection and safety of personal data, then provided written informed consent. The present study was conducted based on the guidelines and regulations.

### Stimuli

**Figure 1:**
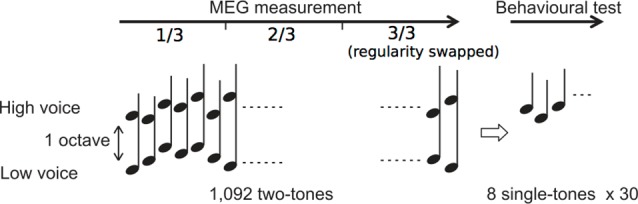
Experimental procedure. Two simultaneous sequences consisting of high- and low-voice sequences were presented during magnetoencephalography (MEG) measurement. After the measurement, behavioral tests were conducted.

We used the same stimuli as those in our previous study (Daikoku and Yumoto, 2017). The eight complex tones consisted of four high and low pitches each based on a five-tone equal temperament (100 × 2^(n - 1)/5^ Hz, high: *n* = 11–14: 400, 459, 528, and 606 Hz; low: *n* = 1–4: 100, 115, 132, and 152 Hz; duration 350 ms with rise/fall of 10/150 ms; 80 dBSPL intensity and binaural presentation). The sequence consisted of 1,092 repetitions of two-tone chords (SOA = 500 ms), each of which consisted of a high and low pitches within which the intervals were separated by more than one octave (Figure 1).

**Figure 2:**
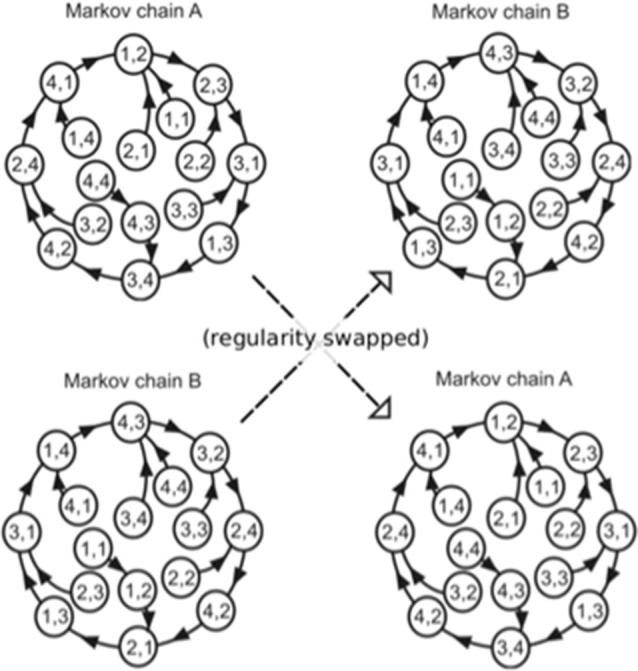
The Markov models used in the present study (Daikoku and Yumoto, 2017). The paired digits in the circles represent two successive tones in the stimulus sequence. The distinct two Markov chains (A,B) were used in each of the low and high voices, and the use of Markov chains was counterbalanced across participants. The solid arrows represent transitions from each state with a high probability (80%). The remaining possible transitions from each state to the other three states occurred with a low probability (6.67% each). In the last third of the sequence, the Markov models controlling sequential regularity of the low and high voices were exchanged.

The order in which the high and low pitches was defined separately based on a second-order Markov model (Markov, 1971, reprinted) with the constraint that the probability of a forthcoming tone was statistically defined (80% for a tone; 6.67% for the other three tones) by the last two successive tones (Figure 2). In the last third of the sequence, however, the Markov models controlling sequential regularity of the low and high voices were exchanged (Figure 1). The regularities of the Markov models were counterbalanced across subjects.

### Experimental Protocol

Subjects listened to a 1,092-dyad sequence with MEG measurement and took a behavioral test immediately afterward. They were instructed to ignore one sequence but attend to the other. The assignment of the attended and ignored sequences was counterbalanced across the subjects. To distinguish between attended and ignored conditions, a silent period of 500 ms was pseudo-randomly inserted within every set of 40 successive tones in attended sequence. Before the session, the subjects were instructed to raise their right hands at every silent period in attended sequence. Thus by observing that all subjects correctly raised their right hands at every silent period, we were able to confirm that they continually paid attention to only attended sequence.

After the measurement, subjects were presented with 30 series each consisting of eight single tones. Subjects answered whether each eight-tone series sounded familiar or not. The 30 series of eight tones could be classified into three types, and the presentation order was randomized. In 10 series, tone stimuli were sequenced using the Markov model that was applied in the last third of the ignored sequence (tone series A). In an additional 10 series, tone stimuli were sequenced according to the same Markov model as an attended sequence in last third of the sequence (tone series B). In the remaining 10 series, tones were pseudo-randomly ordered (random tone series). The behavioral test was completed within 6 min for each subject.

### Measurement and Data Analysis

Measurement and analysis were conducted as in our previous studies (Daikoku and Yumoto, 2017). Selective response averaging was performed separately for the first, middle, and last thirds of the sequence. Responses to each chord were selectively averaged from the beginning of each first, middle, and last thirds of the sequence. They were also selectively averaged in each dyad stimulus: chord that consisted of two high-TP (i.e., frequent) tones in both attended and ignored sequences, chord that consisted of two low-TP (i.e., rare) tones in both attended and ignored sequences, chord that consisted of a frequent tone in attended sequence and a rare tone in ignored sequence, and chord that consisted of a rare tone in the attended sequence and a frequent tone in the ignored sequence. The averaged responses were filtered offline with a 2–40 Hz band-pass. The baseline for the magnetic signals in each MEG channel was defined by the mean amplitude in the pre-dyad period from −100 to 0 ms. The analysis window was defined as 0–500 ms. In addition to selective averaging, all responses (1,092-dyad stimuli) to the dyads were averaged in each subject, enabling us to evaluate reliability for individual components. Using the averaged responses to all 1,092-dyad stimuli, the P1m, N1m and P2m were separately modeled as single equivalent current dipoles (ECDs) in each hemisphere (Daikoku et al., 2017a). The ECDs were calculated from the averaged responses to all 1,092-dyad stimuli with a goodness of fit above 80% using the 66 temporal channels (44 gradiometers and 22 magnetometers) for each participant. The selected channel areas correspond to our previous studies (Daikoku et al., 2014, 2015, 2016, 2017a). Subjects who demonstrated poor ECD estimation, with a goodness-of-fit below 80% in either the left or right hemisphere, were discarded from further analyses. Consequently, learning effects on the P1m, N1m, and P2m components were studied in 13, 10, and 11 subjects, respectively. Because a lot of the goodness of fit in the ECDs for the N1m and P2m were less than 80%, they were excluded from the analyses in this study.

Using the ECDs, the source-strength for P1 m in each hemisphere were calculated based on selective response averaging. Then, we performed a 3 (portion: first, middle, and last) × 2 (hemisphere: right and left) × 4 (dyad stimulus: chord that consisted of two frequent tones in both attended and ignored sequences, chord that consisted of two rare tones in both attended and ignored sequences, chord that consisted of a frequent tone in attended sequence and a rare tone in ignored sequence, and chord that consisted of a rare tone in the attended sequence and a frequent tone in the ignored sequence) repeated-measures analysis of variance (ANOVA) with peak amplitude and the latency of the source-strength of P1m. Bonferroni-corrected *post hoc* tests were conducted for further analysis. Furthermore, we performed ANOVA with the logit values of the familiarity ratios in behavioral test. Significance levels were set at *p* = 0.05 for all analyses. For further analysis, *post hoc* tests with Bonferroni correction were performed.

## Results

### Behavioral Results

**Figure 3:**
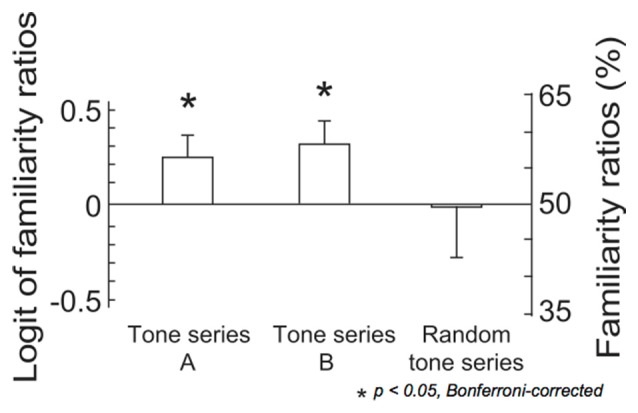
The logit values and percentages of familiarity ratios. In tone series A, tones were sequenced using the constraint that was applied in the last third of the ignored sequence. In tone series B, tones were sequenced using the constraint that was applied in the last third of the attended sequence. In the remaining 10 series, tones were pseudo-randomly sequenced (random tone series). The bars indicate the standard error of the mean. Asterisks indicate significant differences in a pairwise test, (*p* < 0.05, Bonferroni-corrected).

The results of two-tailed *t*-tests indicated that the familiarity ratios were significantly above chance level in both tone series A and tone series B (tone series A: *t*_(14)_ = 2.30, *p* = 0.037, tone series B: *t*_(14)_ = 2.46, *p* = 0.028; Figure 3). The ANOVA detected no significant results.

### MEG Results

**Figure 4:**
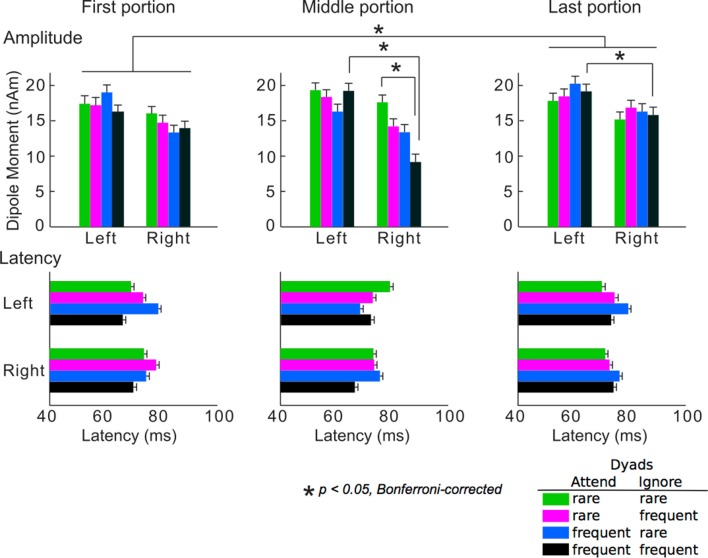
Mean peak amplitudes (upper) and the latencies (lower) of P1m. Green bars: responses to chords consisting of two rare tones in attended and ignored sequences; pink bars: responses to chords consisting of a rare tone in attended sequence and a frequent tone in ignored sequence; blue bars: responses to chords consisting of a frequent tone in attended sequence and a rare tone in ignored sequence; black bars: responses to chords consisting of two frequent tones in attended and ignored sequences. Asterisks indicate significant differences (*p* < 0.05, Bonferroni-corrected).

The averaged peak amplitudes and latencies of P1m responses are shown in Figure 4. The ANOVA detected that the main portion effect on the amplitudes was significant (*F*_(2,24)_ = 3.74, *p* = 0.039). The amplitudes in the last portion were significantly greater than those in the first portion (*p* = 0.049). The hemisphere-stimulus-portion interaction of the amplitudes was significant (*F*_(6,72)_ = 2.32, *p* = 0.042). In the middle and last portions, the amplitudes for the dyads that consisted of two frequent tones were significantly higher in the left than in the right hemispheres (middle: *p* = 0.039, last: *p* = 0.036). In the right hemisphere, the amplitudes for the dyads that consisted of two rare tones were significantly higher than those for the dyads that consisted of two frequent tones in the middle portion (*p* = 0.028). The results were consistent with a body of previous studies on SL: the brain learned TPs of the sequences, predicted a stimulus with a high TP (i.e., frequent stimuli), and inhibited the neural response to the stimuli with a high TP. The SL effects finally represent as a difference amplitudes between the stimuli with high and low TPs (François and Schön, 2011; François et al., 2013, 2017; Paraskevopoulos et al., 2012; Daikoku et al., 2014, 2015, 2016; Koelsch et al., 2016). These SL effects (i.e., difference amplitudes between the stimuli with high and low TPs), however, could not be detected after the Markov chains of the two sequences were exchanged in the last portion. This may suggest that SL effects cannot be retained when sequential regulations are exchanged. There was no significance in latency. No other significant differences were detected.

## Discussion

When the brain encodes the TP distributions of a stimulus sequence, humans expect a probable future stimulus with a high TP and inhibit the neural response to predictable external stimuli. In the end, the effects of SL manifest as a difference in amplitudes between stimuli with lower and higher TPs (Yumoto and Daikoku, 2016; Daikoku, 2018b). In the present study, subjects listened to two simultaneous sequences composed of tones with lower and higher TPs (i.e., rare and frequent tones, respectively). The subjects were instructed to ignore one of the two simultaneous sequences and to attend to the other. Based on the combinations of rare and frequent tones in the two simultaneous sequences, there were four types of dyads: dyads consisting of frequent tones in both sequences, dyads consisting of rare tones in both sequences, dyads consisting of a frequent tone in the attended sequence and a rare tone in the ignored sequence, and vice versa. If subjects were able to perform the SL of two sequences, and simultaneously predict stimuli with high TPs in both sequences, dyads consisting of two frequent tones should generate the lowest-amplitude responses, while those consisting of two rare tones should generate the highest-amplitude responses.

We found that, in the right hemisphere, neural responses to dyads consisting of two rare tones in ignored and attended sequences were significantly greater than those to dyads consisting of two frequent tones in ignored and attended sequences. These results suggested that the subjects were able to learn the statistics of the two sequences simultaneously and that SL of a sequence of two-tone dyads may be right-hemisphere dependent. This result is in agreement with previous studies that have reported the SL effects of single-tone sequences to be right-hemisphere dependent (Roser et al., 2011; Danckert et al., 2012; Shaqiri and Anderson, 2013). The amplitude difference could not be retained after the statistical regularities of the two sequences were exchanged in the last third of each sequence, although the finding that the amplitude in the right hemisphere was lower than that in the left was retained. This may imply that learning effects cannot be retained when sequential regulations in the low and high voices are exchanged. A previous study has suggested that explicit knowledge cannot be transformed into implicit knowledge through practice (Krashen, 1982; Hulstijn, 2002). In contrast, other researchers have claimed that implicit and explicit knowledge can interact with each other (DeKeyser, 2003, 2007; De Jong, 2005; Ellis, 2005, 2009). The present study may imply that implicit and explicit learning can interact with each other, but only barely.

SL is reflected in the early component of P1 (Paraskevopoulos et al., 2012; Daikoku et al., 2016, 2017c) as well as in the late components such as N1, mismatch negativity (MMN), P2, and N400 (Abla et al., 2008; Furl et al., 2011; Daikoku et al., 2014, 2015; Koelsch et al., 2016). It is, however, considered that the SL effect relationship with P1 involves music expertise and specialized training experience (Boutros et al., 1995; Boutros and Belger, 1999; Kisley et al., 2004; Kizkin et al., 2006; Wang et al., 2009). According to a previous study (Adler et al., 1982), earlier auditory responses such as P1 were attributed to parallel thalamo-cortical connections and superior temporal gyrus. Thus, the findings of P1 in the present study can be interpreted as a prediction error suppressed by top-down predictions (Friston, 2005). Further studies are needed to reveal the role of P1 in SL.

Previous studies have suggested that the brain regions and activation patterns engaged during attentional and nonattentional learning might be partially distinct (Curran and Keele, 1993; Rauch et al., 1995; Reber and Squire, 1998; Jimenez and Castor, 1999; Poldrack et al., 2001; Aizenstein et al., 2004; Paradis, 2004; Destrebecqz et al., 2005; Daikoku and Yumoto, 2017). In our recent study, the SL of two simultaneous sequences was facilitated by paying attention to only one sequence and ignoring the other (Daikoku and Yumoto, 2017). This suggests that there is a partially distinct neural basis of attentional and nonattentional SL. In other words, biased attention might be an essential strategy in situations where the learner is exposed to multiple streams of information simultaneously. In this study, we exchanged the Markov model between attentional and nonattentional sequences in the last third of the sequences. We also revealed that the SL of two simultaneous auditory sequences might be right-hemisphere dependent. Learning effects cannot be retained when the tone sequence to which the subject is attending is changed during listening. These results suggest that attentional and nonattentional learning scarcely interact with each other and that there may be a specific cognitive system for nonattentional learning that is independent of attentional learning. As we could not demonstrate a neurological dichotomy between nonattentional and attentional SL due to the methodological limitations of the present study, further studies are needed to examine distinct or common neural mechanisms between attentional and nonattentional learning.

## Author Contributions

The experimental paradigms of the present study were considered by both of the authors. TD made the paradigms, recruited the participants, and collected the data; analyzed all of the data. MY proposed methodologies of MEG/behavioral data analyses and figures in the manuscript. Both of the authors discussed how the results could neurophysiologically and psychologically be interpreted. TD prepared the figures, and wrote the main manuscript text. Then, both of the authors reviewed and revised the manuscript.

## Conflict of Interest Statement

The authors declare that the research was conducted in the absence of any commercial or financial relationships that could be construed as a potential conflict of interest.
